# Oxycodone enhances antitumor effect of paclitaxel on human breast cancer SKBR3 cells *in vitro*

**DOI:** 10.1016/j.clinsp.2024.100458

**Published:** 2024-07-30

**Authors:** Fangfang Liu, Hongmei Yuan, Chenyang Xu, Mingjie Mao, Shanwu Feng

**Affiliations:** Department of Anesthesiology, Women’s Hospital of Nanjing Medical University, Nanjing Women and Children’s Healthcare Hospital, Nanjing, China

**Keywords:** SKBR3 breast cancer, Oxycodone, Paclitaxel, Apoptosis

## Abstract

**Background:**

The influences of Oxycodone (OXY) combined with Paclitaxel (PTX) on breast cancer cells are unclear. The present study aimed to examine the effects of OXY combined with PTX on the proliferation, apoptosis, and migration of human breast cancer SKBR3 cells and the underlying mechanism.

**Methods:**

The proliferation, apoptosis and invasion of SKBR3 cells were assessed by CCK-8, colony formation assay, flowcytometric, Transwell assay and scratch assays, respectively. In addition, Western blotting was used to detect the expression of related proteins in these cells. The autophagic bodies were observed under a transmission electron microscope.

**Results:**

OXY (0.25, 0.5 and 1 mM) significantly inhibited the viability, colony-forming, migration, and invasion of SKBR3 cells as compared to the control group. Furthermore, OXY (0.25, 0.5 and 1 mM) markedly induced the apoptosis of SKBR3 cells and the levels of apoptosis-related proteins. In addition, OXY (0.25, 0.5 and 1 mM) and PTX inhibited the proliferation of SKBR3 cells synergistically as compared to PTX group in vitro. Moreover, OXY (0.25, 0.5 and 1 mM) significantly elevated the PTX-induced apoptosis in SKBR3 cells via downregulating the expression of N-cadherin, Becline-1 LC3-Ⅱ, p-Akt and p-mTOR and upregulating E-cadherin expression. Compared with the control group, OXY (1 mM) treatment induced autophagy in SKBR3 cells.

**Conclusions:**

The present study indicates that OXY can enhance the antitumor effect of PTX on breast cancer in vitro. Hence, the combination of OXY with PTX may serve as a potential strategy for the treatment of breast cancer.

## Highlights

• Oxycodone markedly induced the apoptosis of SKBR3 cells.
• Oxycodone distinguished enhanced antitumor effect of PTX on SKBR3 cells.
• OXY can enhance antitumor effect of PTX on breast cancer *in vitro*.

## Introduction

As the most common cancer in females, breast cancer is considered the leading cause of cancer-related death in women.^1^ Up to now, surgery is still the most commonly used therapy for breast cancer, and chemotherapy, radiotherapy, endocrinology, immunity and targeted therapy may serve as adjunctive therapies.^2^^,^^3^ In decades, great progress has been made in the therapy of breast cancer, however, the perioperative analgesia, treatment of advanced cancer pain and prevention of tumor recurrence have been clinical challenges in clinical practice.^4^ Therefore, comprehensive consideration of treatment plans is critical in the management of breast cancer.

Oxycodone (OXY) is a semisynthetic opioid that can bind to both μ-and κ-opioid receptors derived from the Bain.^5^ Increasingly, studies have used OXY in the treatment of moderate to severe perioperative pain and chronic cancer pain. Studies have indicated that OXY can promote or prevent tumor growth and metastasis.^6^^,^^7^ Paclitaxel (PTX) is an effective drug for the chemotherapy of cancers and has been widely applied in clinical. However, the severe side effects and multiple drug resistance greatly limit the wide clinical application of PTX. Hence, the present study aimed to investigate the antitumor effects of PTX combined with OXY on human breast cancer SKBR3 cells *in vitro*, and explore the potential mechanism.

## Materials and methods

### Drugs

OXY was obtained from Mundipharma Pharmaceutical Co. Ltd. (Cambridge, UK) and PTX from Yangtze River pharmaceutical group Co., Ltd. (Jiangsu, China).

### Cell culture and treatment

Human breast cancer SKBR3 cells were provided by the Department of Oncology, Jinling Hospital, General Hospital of Eastern Theater Command (Nanjing, China). These cells were maintained in Dulbecco's modified Eagle's medium (DMEM, KeyGEN BioTECH, China) with the addition of 10 % fetal bovine serum (FBS, Gibco, USA) at 37°C and 5 % CO_2_.

### CCK-8 proliferation assay

The cell viability was examined by Cell Counting Kit-8 (CCK-8, APExBIO, USA) according to the manufacturer's protocols. Briefly, cells were seeded into 96-well plates at a density of 3 × 10^3^ cells per well and then incubated at 37°C overnight. Then, these cells were treated with OXY (0.025, 0.05, 0.1, 0.25, 0.5, 1 and 2 mM) and/or PTX (2, 4, 8, 16, 32 and 64 μM) for 24h or 48h. There were at least 5 wells in each group, and the measurement was repeated at least 3 times. In brief, 10 μL CCK-8 solution was dropped to each well, followed by incubation for 1h at 37°C. The cell viability was evaluated by examining the absorbance at 450 nm.

### Colony formation assay

Cells were seeded into 6-well plates with a density of 800 cells per well, and subsequently incubated with OXY (0.25, 0.5 and 1 mM) and/or PTX (8 μM) for 8‒10 days. Then, cell colonies were fixed by the 4 % paraformaldehyde for 15 min and subsequently stained with 0.1 % crystal violet (Beyotime, Jiangsu, China) for 30 min. These cells were photographed using a microscope (Olympus, Tokyo, Japan). Colonies containing at least 50 cells were scored.

### Wound scratch assay

Three lines were delineated at the back of a 6-well plate through a marker. These transverse lines were uniformly drawn at a distance of 0.5‒1 cm between lines with the assistance of a ruler, and the lines passed through the wells. Cells were incubated in 6 well plates. When the cell's monolayer reached 90 % confluence, each microplate was scratched in three straight lines with a pipette tip. After washing with Phosphate-Buffered Saline (PBS), the medium was changed to the medium containing 1 % FBS and OXY and/or PTX at different concentrations. Images were captured using a DSC-HX1 digital camera (Sony Corporation, Tokyo, Japan) at each time point (0h, 24h and 48h).

The migration rate was measured by capturing images at different time points using a digital camera. The width of the wound was measured at the widest point, and the migration rate was calculated as the percentage of the initial wound width that had healed at each time point. At least three independent experiments were performed for each group.

### Transwell migration and invasion assays

Transwell assays were conducted using 24-well plates with the insertion of 8 μm pore-size Transwell (Corning Incorporated, Corning, USA). Cells treated with OXY (0.25, 0.5, 1 mM) and/or PTX (8 μM) were counted and subsequently resuspended in serum-free Dulbecco's Modified Eagle Medium (DMEM). 100 μL DEME medium containing 5×105 cells in each group were added to each upper chamber for migrating ability assessment. The lower chambers were added with 800 μL medium containing 20 % FBS. The cells migrated to the lower chamber and were fixed for 5 min by 4 % paraformaldehyde following incubation for 24h, then treated with methanol for 10 min and then 0.1 % crystal violet for 15 min. The cells were counted at a magnification of ×100 under a light microscope (Sony Corporation, Tokyo, Japan). The cell number was evaluated by Image J software and expressed as the average cell number in each field (Rawak Software, Inc. Germany).

For invasion assays, 50 μL matrix glue with Matrigel (BD Biosciences, USA; 1:8) was added into the upper chambers before adding cells into the upper chambers. Then, the plates were incubated at 37°C for 5h and the following procedures were the same to those above mentioned.

### Detection of cell apoptosis by flow cytometry

SKBR3 cells (5×105 cells/well) were seeded into 6 well plates and then incubated at 37°C overnight. Cells were pre-treated with OXY (0.25, 0.5 and 1 mM) and/or PTX (10 μM) for 24h. According to the manufacturer's protocols, cells were collected and resuspended in 195 μL binding buffer. Subsequently, 5 μL Annexin V-FITC (Beyotime, Jiangsu, China) and 5 μL propidium iodide (PI, Beyotime, Jiangsu, China) were added to each cell suspension, followed by 20 min incubation at room temperature in dark. Cell apoptosis was evaluated by flow cytometry (BD Biosciences, USA).

### Western blotting

After different treatments, cells were incubated with RIPA buffer at 4°C for 10 min. Total protein was collected through the 12,000× g centrifugation for 30 min (4°C) and quantified with a BCA kit (NCM Biotech, Suzhou, China). Proteins were separated by 10 % SDS-PAGE and transferred onto PVDF membranes (EMD Millipore, Billerica, MA). After 1h block with 5 % fat-free milk, the membranes were incubated with primary antibodies overnight at 4°C. Then the secondary antibodies were applied in the incubation with the membranes for 1h at room temperature after washing with TBST. The protein bands were visualized with ECL reagent (Beyotime, Jiangsu, China) and then quantified.

The primary antibodies used in present study were as follows: anti-Bcl2 (1:1000, ab196495), anti-Bax (1:2000, ab3191), anti-E-cadherin (1:2000, ab238099), anti-N-cadherin (1:2000, ab254512), anti- GAPDH (1:5000, ab181602), anti-PI3K (1:5000, ab32089), anti-AKT (1:5000, ab81283), anti-mTOR (1:5000, ab32028), anti-LC3-Ⅱ(1:5000, ab222776), anti-Beclin1(1:5000, ab207612), anti-p-AKT (1:5000, ab8805) and anti-p-mTOR (1:5000, ab84400) (Abcam, UK).

### Transmission electron microscopy

Cells were collected by centrifugation at 1000 R /min for 5 min, and subsequently incubated with 2.5 % glutaraldehyde at 4°C for 24h. Then, the cells were post-fixed in 1 % osmium tetroxide solution for 1h, dehydrated in 90 % acetone for 15 min and in 100 % acetone for 10 min (3 times), and finally embedded in Epon LX-112 resin. The blocks were sliced into ultrathin slices and double-stained with uranyl acetate and lead citrate. Autophagic bodies were observed under a transmission electron microscope (IEM-1200exv).

### Statistical analysis

There were at least three independent experiments in each group and all the data are expressed as mean ± standard deviation. Student's *t*-test was conducted to measure the comparison between two groups, and a one-way analysis of variance followed by Dunnett's test was applied in multiple groups. A value of p < 0.05 was considered statistically significant.

## Results

### OXY enhances the effect of PTX on the proliferation of SKBR3 cells

**Figure 1:**
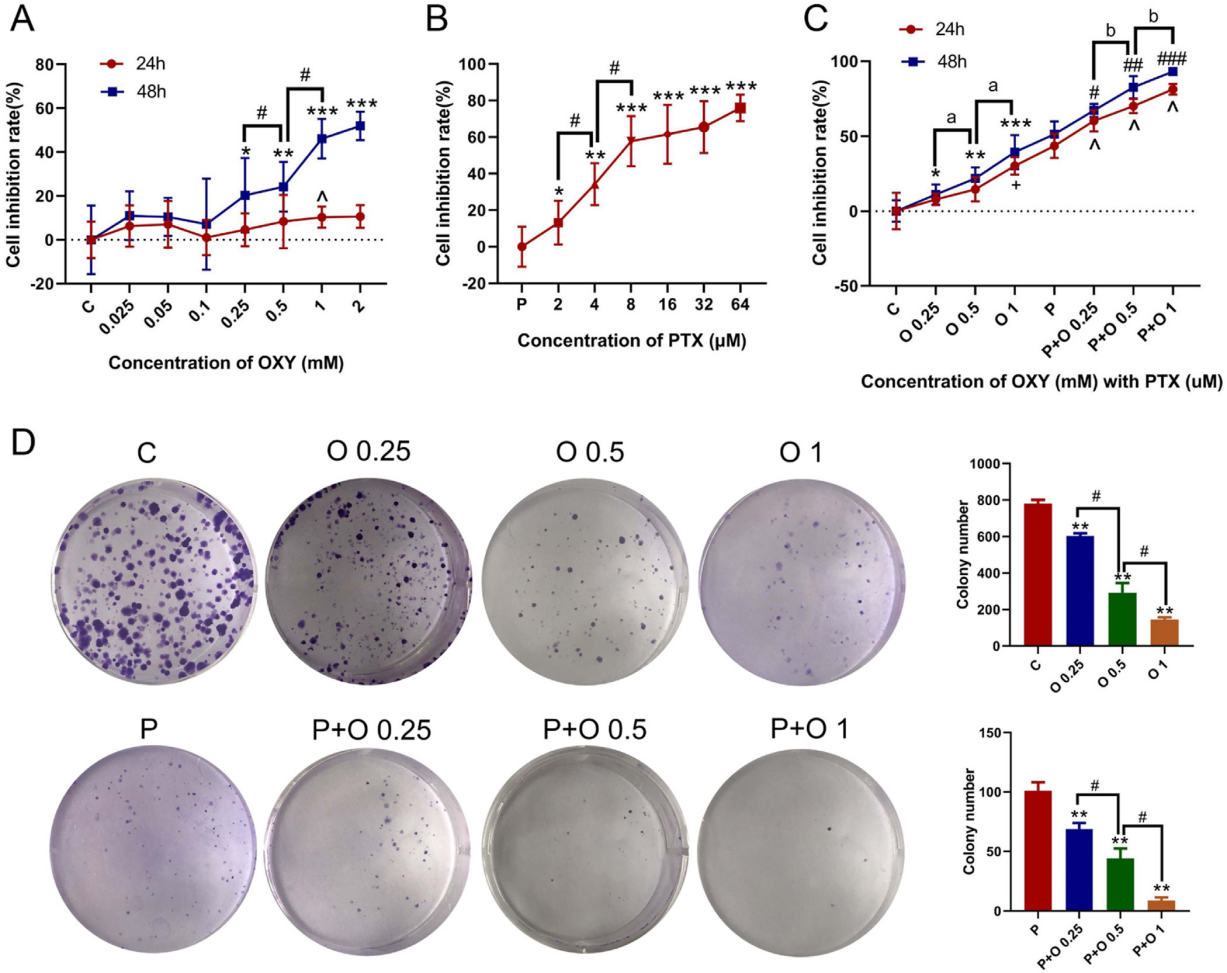
OXY inhibited the viability of SKBR3 cells and the combination of OXY with PTX synergistically inhibited the proliferation of SKBR3 cells. (A) Cell viability was detected by CCK-8 assay in SKBR3 cells treated with OXY (0.025, 0.05, 0.1, 0.25, 0.5, 1 and 2 mM) for 24h and 48h. * p < 0.05 and ^ p < 0.05 vs group C. # p < 0.05, OXY (0.25, 0.1 mM) group vs OXY (0.5 mM) group. (B) Cell viability was detected by CCK-8 assay in SKBR3 cells treated with PTX (2, 4, 8, 16, 32 and 64 μM) for 24h. * p < 0.05 vs. group P. # p < 0.05, PTX (2, 8 μM) group vs. PTX (4 μM) group. (C) Cell viability was detected by CCK-8 assay in SKBR3 cells treated with PTX (8 μM) and OXY (0.25, 0.5, 1 mM) for 24h and 48h. * p < 0.05, + p < 0.05 vs. group C. # p < 0.05, ^ p < 0.05 vs. group P. ^a^ p < 0.05, OXY (0.25, 0.1 mM) group vs. OXY (0.5 mM) group. ^b^ p < 0.05, P+O 0.25 group and P+O 1 group vs. P+O 0.25 group. (D) Colony formation capability of SKBR3 cells was assessed after treatment with OXY (0.25, 0.5, 1 mM) for 48h, and/or PTX (8 μM) for 24h. * p < 0.05 vs. group C; # p < 0.05 vs. group P. At least three independent experiments in each group. Abbreviations: P, Paclitaxel; C, Control; O, Oxycodone.

To investigate the effects of OXY and/or PTX on the malignant biological behaviors of SKBR3 cells, the viability and colony-forming ability of SKBR3 cells were evaluated by CCK-8 and colony formation assays. OXY (0.25, 0.5 and 1 mM) inhibited the proliferation of SKBR3 cells in a dose-dependent manner within 48h (p < 0.05, Fig. 1A). Meanwhile, 8 μM PTX restrained the proliferation of SKBR3 cells significantly and dementated about 50 % growth inhibition (p < 0.05). However, increasing the concentration of PTX had no significant effect on the inhibition rate at 24h (Fig. 1B). Therefore, 8 μM PTX was used in the following experiments. Moreover, OXY (0.25, 0.5 and 1 mM) markedly augmented the outstanding antiproliferative effect of PTX on the SKBR3 cells, which was dose-dependent (p < 0.05, Fig. 1C). Similarly, colony formation assay revealed that OXY (0.25, 0.5 and 1 mM) remarkably decreased the clonogenic survival of SKBR3 cells within 48 h, and OXY (1 mM) significantly reduced the number of clones as compared to OXY (0.25 and 0.5 mM) (Fig. 1D, p < 0.05). As shown in Figure 1D, OXY (0.25 and 0.5 mM) and PTX (8 μM) synergistically decreased the proliferation of SKBR3 cells within 24h (p < 0.05).

### OXY enhanced the effect of PTX on the Migration and Invasion of SKBR3 Cells

**Figure 2:**
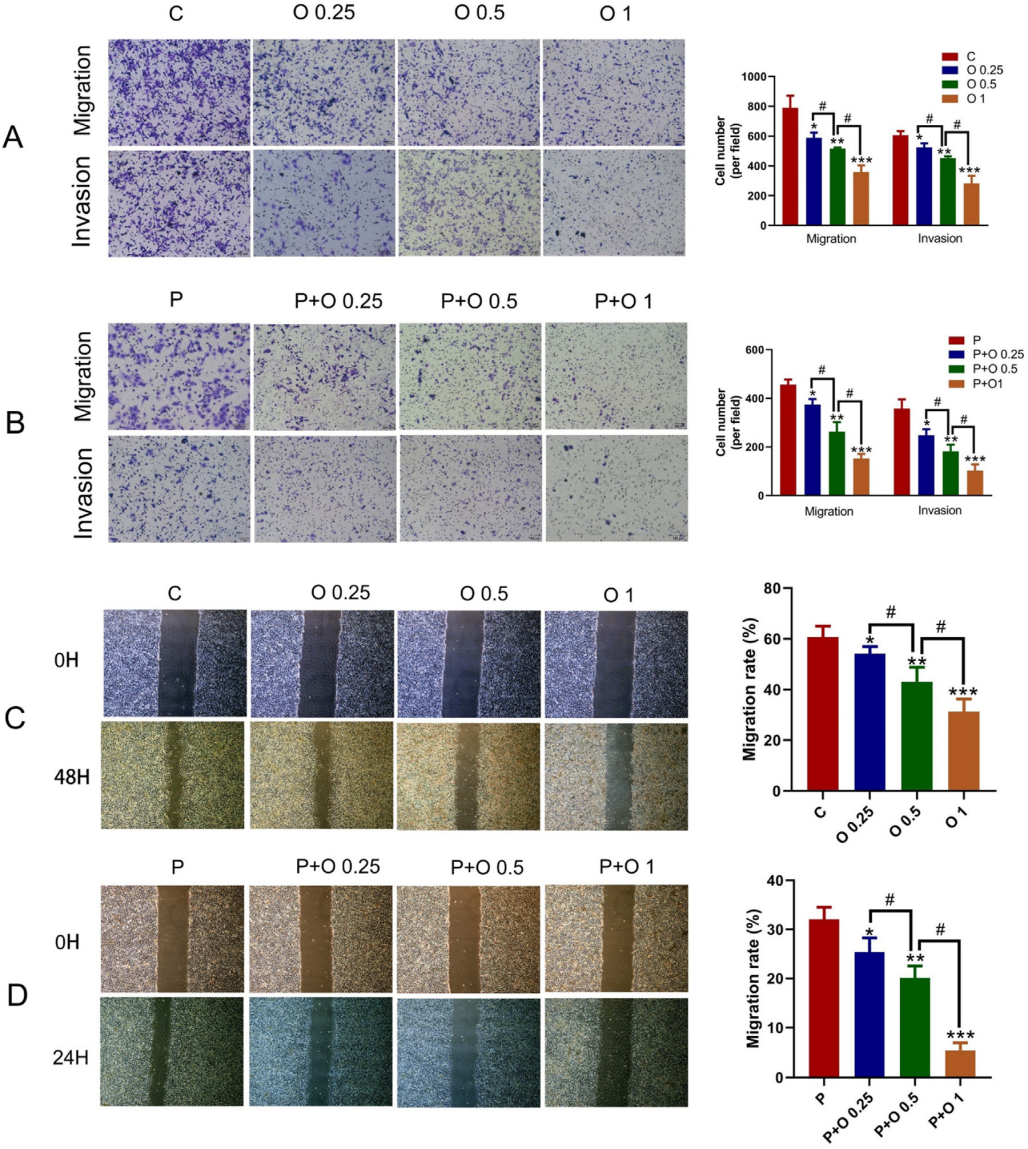
OXY inhibited the migration and invasion of SKBR3 cells and the combination of OXY with PTX synergistically promoted these effects on SKBR3 cells. (A) The migration and invasion of SKBR3 cells were detected by Transwell assay after treatment with oxycodone (0.25, 0.5, 1 mM) for 48h. (B) Combination of OXY (0.25, 0.5, 1 mM) with PTX synergistically inhibited the migration and invasion of SKBR3 cells within 24h. (C) The migration and invasion of SKBR3 cells were detected by wound scratch assay after treatment with OXY (0.25, 0.5, 1 mM) for 48h. (D) The migration and invasion of SKBR3 cells was detected by wound scratch assay after treatment with OXY (0.25, 0.5, 1 mM) and PTX for 24h. * p < 0.05 vs. group C and P. # p < 0.05 vs. group P. # p < 0.05, O 0.25 group and O 1 group vs. O 0.5 group, P+ O 0.25 group and P+ O 1 group vs. P+ O 0.25 group. At least three independent experiments in each group. Abbreviation: P, Paclitaxel; C, Control; O, Oxycodone.

The effects of OXY (0.25, 0.5 and 1 mM) and/or PTX (8 μM) on the migration and invasion of SKBR3 cells were further examined by Transwell and wound scratch experiments. OXY treatment significantly diminished the migration and invasion of SKBR3 cells in a dose-dependent manner within 48h (p < 0.05, Fig. 2A, B). Additionally, OXY enhanced the inhibitory effect of PTX on the SKBR3 cells within 24h, and OXY at the highest concentration (1 mM) achieved the most obvious effect (p < 0.05). Wound scratch assay revealed that OXY significantly attenuated the migratory ability of SKBR3 cells in a dose-dependent manner (p < 0.05, Fig. 2C); whereas the migratory ability of SKBR3 cells further reduced in a dose‑dependent manner in the OXY plus PTX group (Fig. 2D, p < 0.05). In other words, the migration distance in the OXY (1 mM) group was significantly longer as compared to the control. Moreover, the distance in the OXY (1 mM) plus PTX group was markedly longer than in the PTX group (p < 0.05).

### OXY enhanced PTX-induced apoptosis of SKBR3 cells

**Figure 3:**
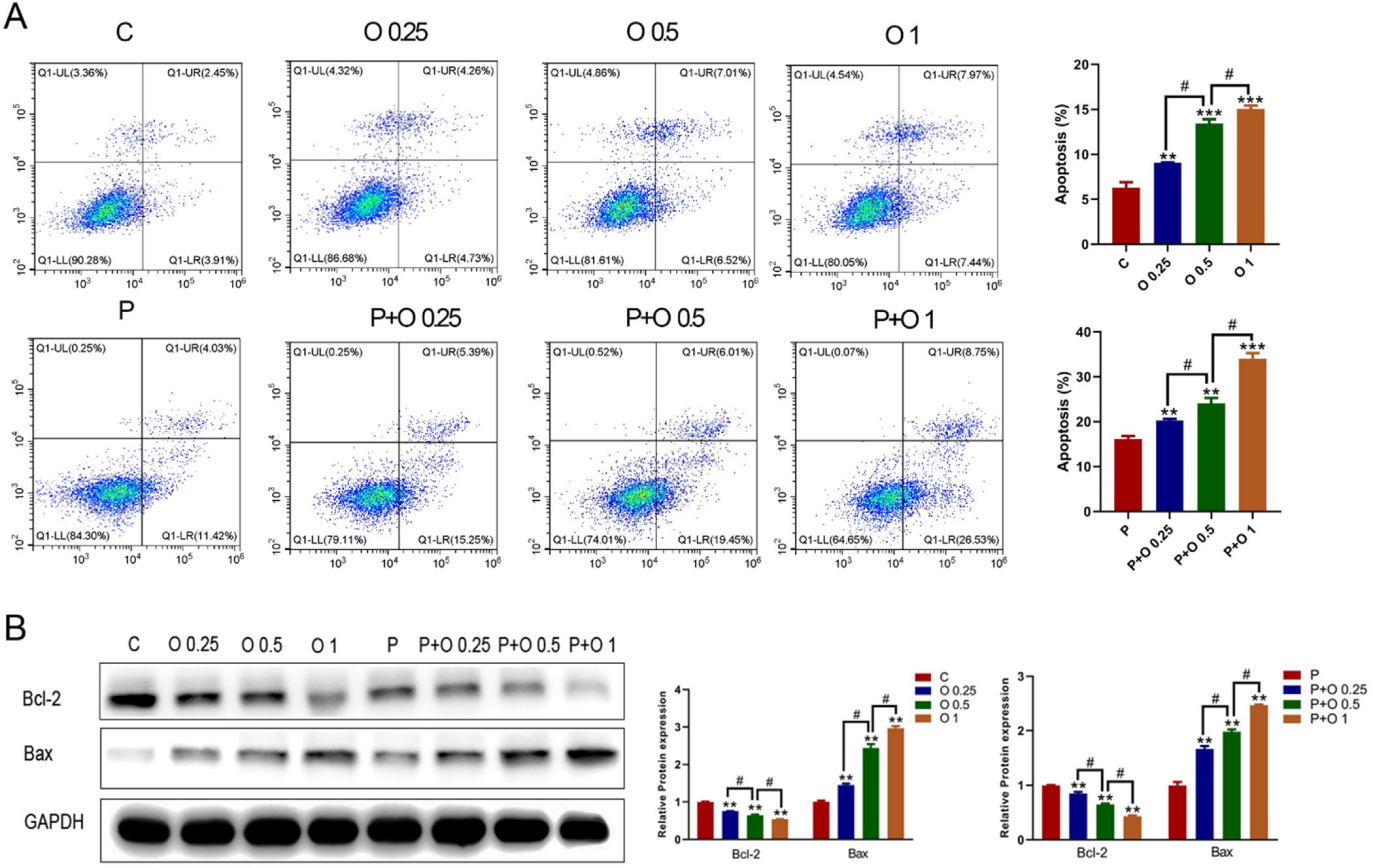
OXY enhanced PTX-induced apoptosis in SKBR3 cells. (A) Cells were treated with OXY (0.25, 0.5, 1 mM) for 48h, and with OXY plus PTX (8 μM) for 24h. Apoptotic cells were detected after Annexin V and PI double staining. The apoptotic rate was calculated. * p < 0.05 vs. group C and P. (B) The expression of apoptosis-related proteins (Bcl-2 and Bax) was detected by Western blotting. * p < 0.05 vs. group C and P. # p < 0.05, O 0.25 group and O 1 group vs. O 0.5 group, P+ O 0.25 group and P+ O 1 group vs. P+ O 0.25 group. At least three independent experiments in each group. Abbreviation: P, Paclitaxel; C, Control; O, Oxycodone.

The apoptotic rate significantly increased after OXY treatment (p < 0.05; Fig. 3A). Moreover, the antiproliferative effect of PTX was significantly augmented in the presence of OXY, compared with cells treated with PTX alone (p < 0.05; Fig. 3A). Besides, PTX-induced cell apoptosis was enhanced in the presence of OXY (p < 0.05; Fig. 3A).

As shown in Fig. 3B, the Bcl2 expression decreased, while Bax expression increased after OXY treatment (p < 0.05). This effect of OXY (1 mM) was the most evident in the OXY group. As expected, Bax expression in cells further increased, while Bcl-2expression further decreased after treatment with PTX and OXY as compared to cells treated with PTX (Fig. 3B). These findings implied that OXY enhanced PTX-induced apoptosis in SKBR3 cells.

### OXY promoted the PTX-induced inhibition of epithelial mesenchymal transition in SKBR3 cells

**Figure 4:**
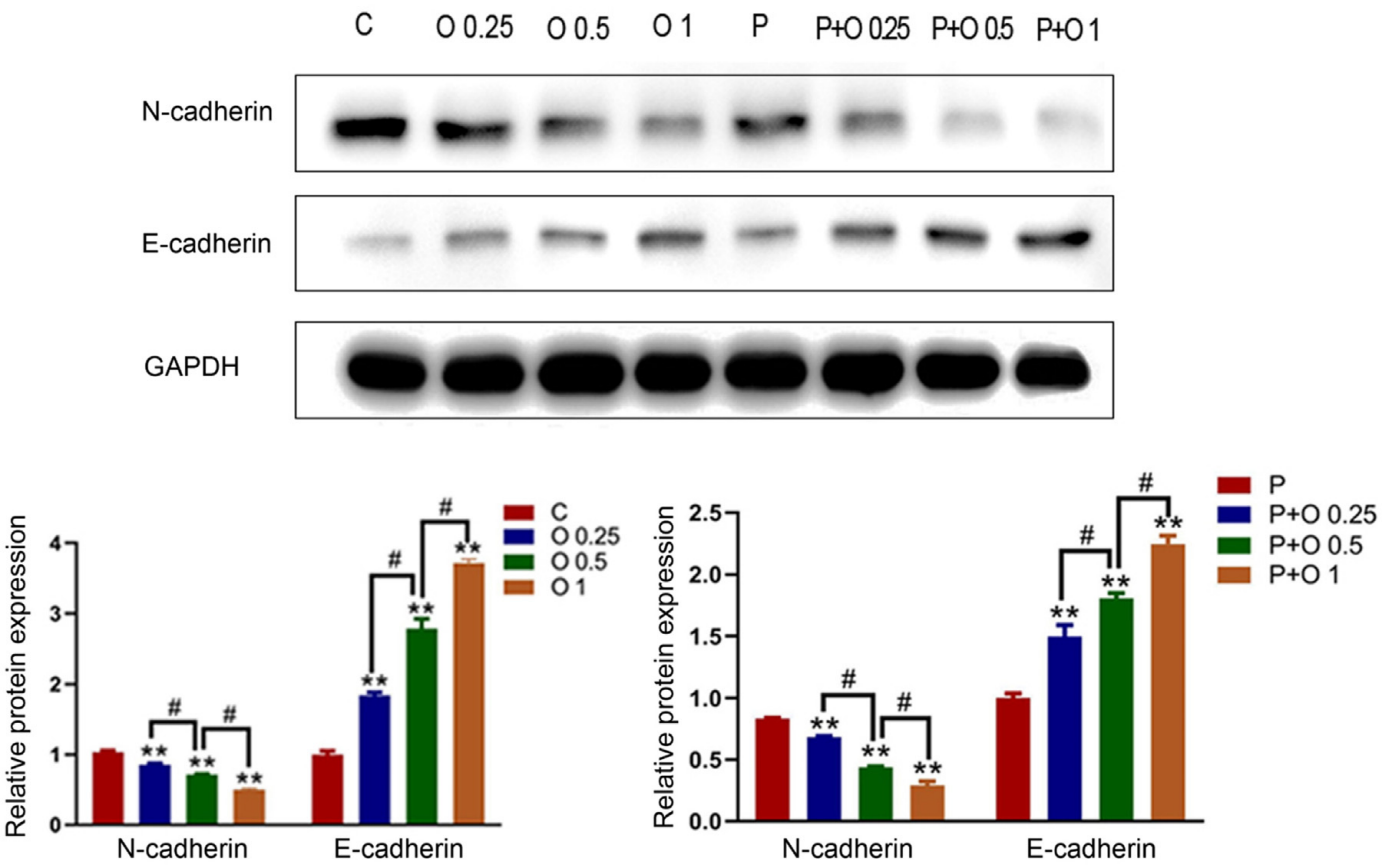
OXY and OXY plus PTX inhibited EMT in SKBR3 cells. The expression of EMT-related proteins (D-cadherin and E-cadherin) was detected by Western blotting. * p < 0.05 vs. group C and P. # p < 0.05, O 0.25 group and O 1 group vs. O 0.5 group, P+ O 0.25 group and P+ O 1 group vs. P+ O 0.25 group. At least three independent experiments in each group. Abbreviation: P, paclitaxel; C, control; O, oxycodone.

Studies have shown that Epithelial Mesenchymal Transition (EMT) is closely related to the tumor metastasis and invasion.^3^ Therefore, EMT-related proteins were assessed in the present study. As compared to cells treated with PTX alone and the control group (p < 0.05, Fig. 4), OXY increased the expression of epithelial marker E-cadherin while decreasing the expression of mesenchymal marker N-cadherin in a dose-dependent manner These results demonstrated that OXY inhibited EMT to compromise the migration and invasion of SKBR3 cells and OXY exerted synergistic effect with PTX.

### OXY combined with PTX upregulated expression of autophagy-related proteins and decreased PI3K/AKT/mTOR-related molecules in SKBR3 Cells

**Figure 5:**
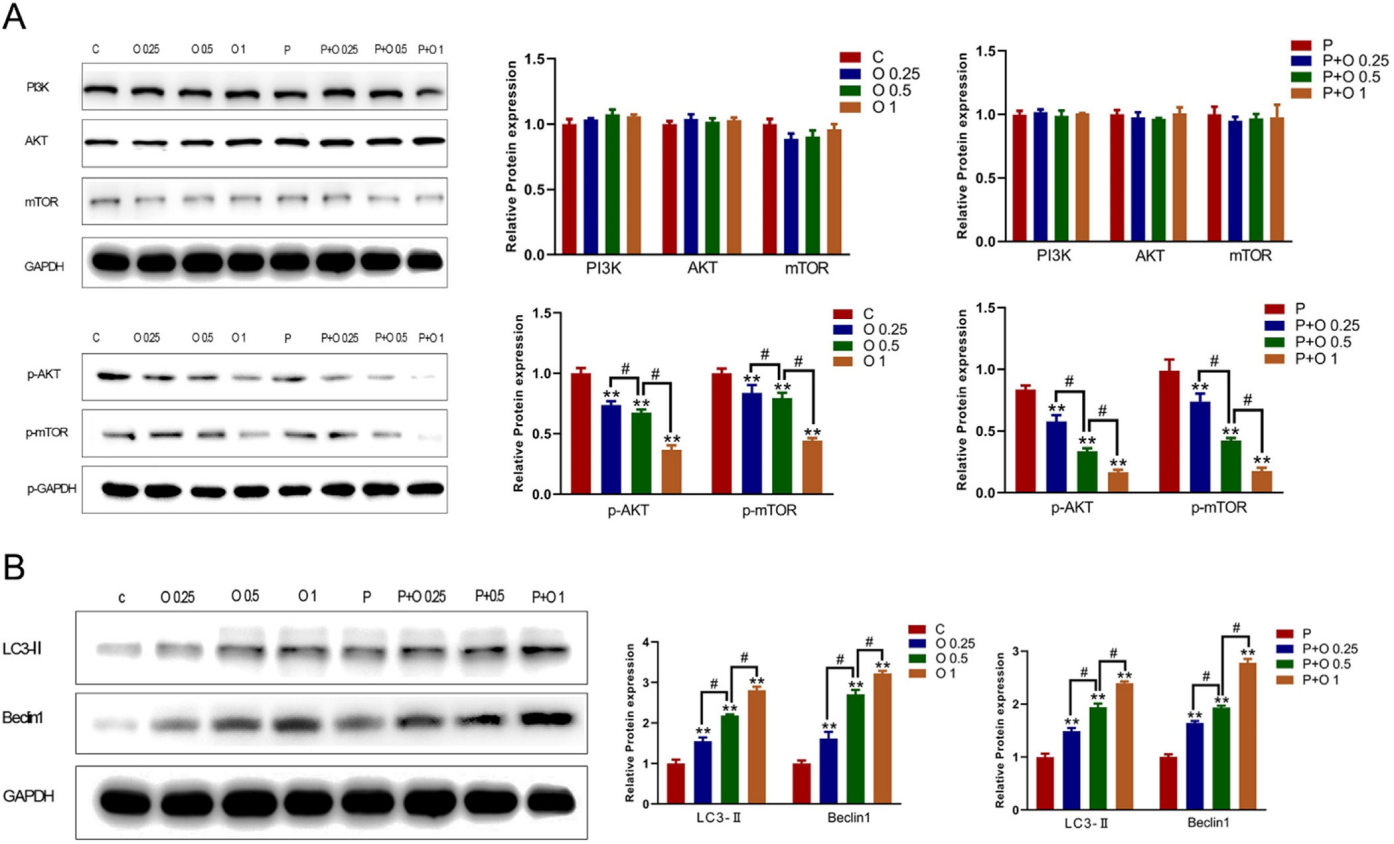
OXY and the combination of OXY with PTX upregulated the expression of autophagy-related proteins and decreased the expression of PI3K/AKT/mTOR-related proteins in SKBR3 cells. (A) The expression of PI3K/AKT/mTOR signaling pathway related proteins (p-AKT and p-mTOR) was detected by Western blotting after different treatments. (B) The expression of autophagy-related proteins (LC3-Ⅱand Becline-1) was detected by Western blotting after different treatments. * p < 0.05 vs. group C and P. # p < 0.05, O 0.25 group and O 1 group vs. O 0.5 group, P+ O 0.25 group and P+ O 1 group vs. P+ O 0.25 group. At least three independent experiments in each group. Abbreviation: P, Paclitaxel; C, Control; O, Oxycodone.

To explore the mechanisms by which OXY and/or PTX restrained the proliferation and enhanced apoptosis of SKBR3 cells, molecules in the PI3K/AKT/mTOR signalling pathway and autophagy were detected by Western blotting. The protein expression of LC3-Ⅱ and Becline-1 (two markers of autophagy) was detected. OXY treatment significantly diminished the levels of p-AKT and p-mTOR (p < 0.05, Fig. 5A) in a dose-dependent manner without affecting the expression of PI3K, AKT and mTOR in SKBR3 cells in comparison with cells treated with PTX alone and cells in the control group. In contrast, the expression of LC3-Ⅱ and Becline1 markedly increased by OXY and/or PTX (Fig. 5B). These findings confirmed that OXY inhibited the phosphorylation of PI3K/AKT/mTOR signalling pathway and affected the process of autophagy.

### OXY and combination of OXY with PTX promote SKBR3 cells autophagy

**Figure 6:**
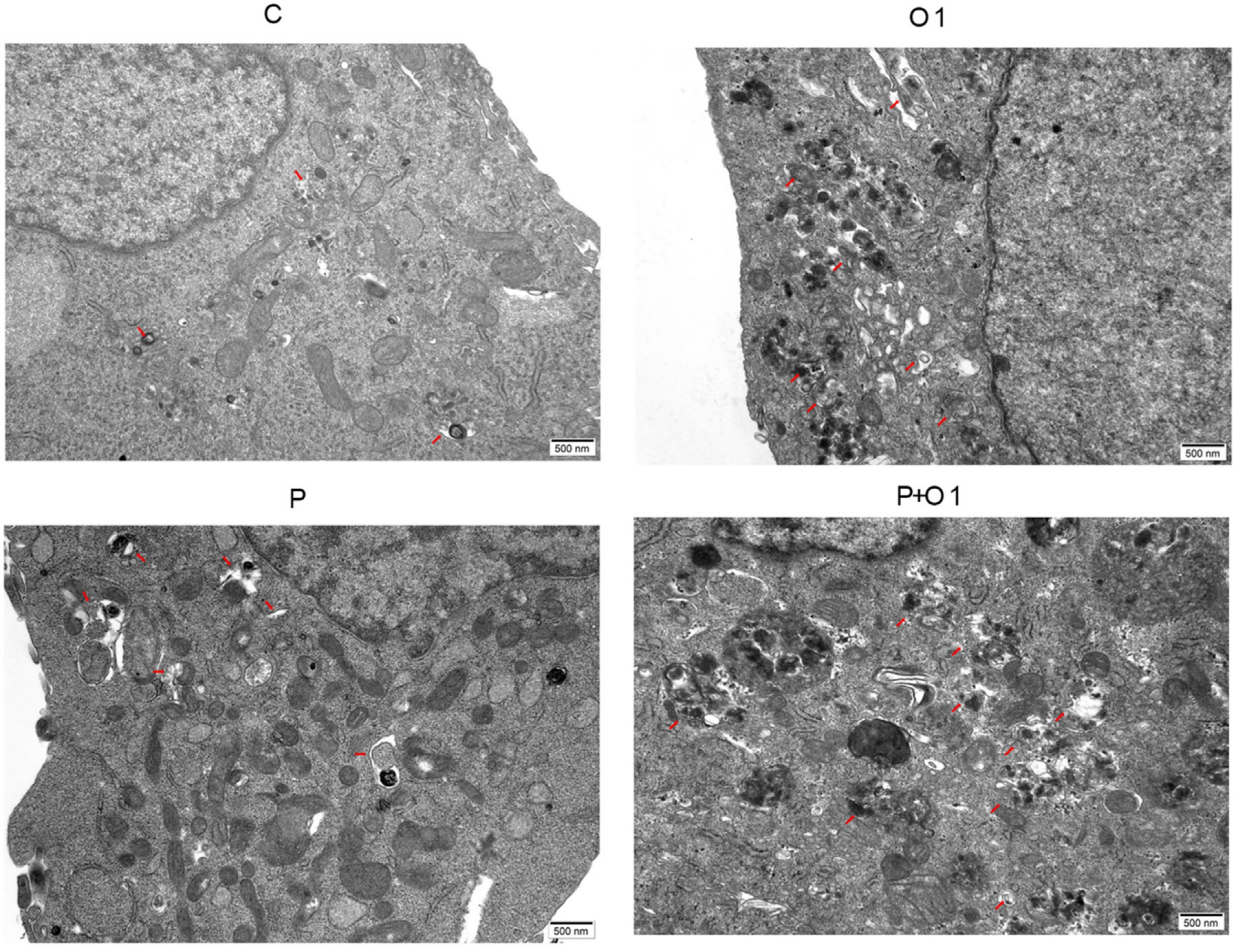
OXY and the combination of OXY with PTX promoted autophagy in SKBR3 cells (TEM). Red arrow: autophagic bodies. Abbreviation: P, Paclitaxel; C, Control; O, Oxycodone.

Autophagy plays an important role in the cancer progression, and Transmission Electron Microscope (TEM) is the gold standard for the detection of autophagy currently. The structural morphology and number of autophagosomes and autolysosomes in cells can be observed by TEM. As displayed in Figure 6, SKBR3 cells in the control group were immature, with fewer cell connections, and no autophagosomes and autolysosomes formed. After treatment with PTX or OXY (1 mM), the proportion of cell cytoplasm increased, vesicles with double-layer membrane structure appeared in the cytoplasm with a size of about 500 nm, and cells appeared autophagosome-like structures. Moreover, treatment with PTX and OXY (1 mM) further enhanced the autophagy of SKBR3 cells.

## Discussion

Breast cancer is the mainly malignancy in women worldwide, and its morbidity and mortality are still rising. Surgery is still the therapy of choice for breast cancer in these patients. Recently, whether perioperative pharmacotherapy affects the recurrence and metastasis of cancers and promotes the occurrence and development of tumours has attracted extensive attention.^1^, ^2^, ^3^

Opioids and anticancer drugs are the most commonly used drugs in the treatment of cancer patients. Generally, both opioids and anticancer drugs are simultaneously administered to patients in clinical practice, especially those with cancer metastasis.^5^, ^6^, ^7^ OXY, a semisynthetic μ-and κ-opioid dual receptor agonist, has been extensively used for the anesthetic pre-medication and the pain management caused by cancer metastasis. Currently, evidence shows that OXY can affect multiple processes in the cancer progression, including immune function, angiogenesis, apoptosis and invasion.^4^^,^^5^ PTX is an effective drug used for the chemotherapy of cancers and has been extensively applied in the clinical therapy of breast cancer. The side effects and drug resistance caused by high-dose and long-term paclitaxel treatment have significantly limited its wide use in clinical practice.^8^ In recent years, the combination therapy of drugs has become an effective strategy to reduce the side effects and improve the efficacy in the treatment of cancers.^9^^,^^10^ Hence, the combination of OXY with PTX may be a potential and appropriate treatment for cancer. To investigate the optimal concentration and the synergistic effects of OXY and PTX, SKBR3 cells were treated with OXY and/or PTX, and their biological behaviours were further investigated. The optimal concentration of OXY was examined by CCK-8 assay (Fig. 1A and B). In the present study, the concentration of OXY ranged from 0 to 2 mM and that of PTX ranged from 0 to 64 μM. The results indicated OXY at < 0.25 mM was unable to reduce cell survival, and OXY at 0.25‒2 mM could exert inhibitory effects on the SKBR3 cells after treatment for 24‒48h as compared to control group. CCK-8 assay, and colony formation assay indicated that OXY (0.25, 0.5 and 1 mM) significantly inhibited the proliferation, migration, and invasion of SKBR3 cells. OXY (0.25, 0.5 and 1 mM) significantly induced the apoptosis of SKBR3 cells on flow cytometry and regulated the expression of Bcl2 and Bax in a concentration-dependent manner. This was consistent with previous findings that OXY could synergistically inhibited the growth of lung and breast cancer^6^^,^^11^ Meanwhile, the present results suggested that the combination treatment significantly improved the antitumor effect of PTX.

Accumulating evidence demonstrates that EMT, PI3K/Akt/mTOR signalling pathway, and autophagy play important roles in the tumor progression, metastasis, and chemoresistance.^9^^,^^12^^,^^13^ Among metastasis process, EMT transforms adherent epithelial cells into highly mobile mesenchymal cells. Then, the migrating and invading abilities of cancer cells are continuously enhanced. E-cadherin is considered as the characteristic marker in epithelial cells which locating on the parts of adhesion junction and basolateral plasma membrane.^14^ N-cadherin is mainly expressed in cells from mesenchymal origin which was closely related to the invasiveness of cancer cells.^14^ Few studies have reported the effect of opioids on the EMT of SKBR3 cells. The present results showed that OXY (0.25, 0.5 and 1 mM) elevated the expression of E-cadherin and decreased N-cadherin. This indicates that the effects of OXY (0.25, 0.5 and 1 mM) on the migration and invasion of breast cancer cells are partly associated with the inhibition of EMT.

PI3K/Akt/mTOR signalling is a key regulator of cellular events such as growth, proliferation, survival and invasiveness in cancers,^13^ and has become an attractive target in the therapy of breast cancer. Evidence has showed that morphine can activate the downstream signalling pathway of AKT-mTOR to promote the proliferation, migration, and invasion of cells.^9^ However, Helmy et al report that PI3K/AKT pathway was involved in the tramadol induced apoptosis of liver HepG2 cells.^14^ In this study, the expression of molecules in the PI3K/Akt/mTOR signalling pathway was further detected in SKBR3 cells treated with OXY or/and PTX. The present results showed that OXY significantly diminished the expression of p-AKT and p-mTOR without affecting the expression of PI3K, AKT and mTOR in SKBR3 cells. These indicate that OXY restraints the malignant behaviours of SKBR3 cells through the phosphorylation of molecules in the PI3K/AKT/mTOR signalling pathway, rather than regulating the expression of these proteins. The present results support OXY as a candidate for the clinical therapy towards breast cancer SKBR3 cells.

Increasing studies have indicated that autophagy functions a crucial role in the pathogenesis of cancers and may be an effective target in the treatment of cancers. LC3, Beclin1 and p62 are autophagy related proteins, which have been considered as independent biomarkers for predicting overall survival and progression-free survival in cancer patients.^9^^,^^15^ LC3A is divided into LC3A, LC3B and LC3C; among them, LC3B is most closely related to autophagy. LC3 is involved in all stages of autophagy. When autophagy occurs, LC3-Ⅰ is activated and binds to phosphatidylethanolamine, and then it converts into LC3-Ⅱ. LC3-Ⅱ may promote the formation and maturation of autophagy and aggregate on autophagy bodies.^9^^,^^15^^,^^16^ Beclin1 is an essential molecule for autophagy, which plays a vital role in the occurrence and development of tumours. Beclin1 expression tends to increase during autophagy.^15^^,^^16^ Some studies have indicated that autophagy participated in the proliferation and apoptosis of cancer cells.^16^^,^^17^ This study confirmed that OXY (0.25, 0.5 and 1 mM) induced the autophagy of SKBR3 cells by upregulating the levels of autophagy related proteins LC3-Ⅱ and Beclin1 and further promoted the autophagy inducing ability of PTX. On TEM, the number and size of autophagic bodies and autophagy vesicles in the OXY (1 mM) group were significantly different from those in the control/PTX group. Above findings indicate that autophagy, EMT and PI3K/Akt/mTOR signalling are involved in the pathogenesis of breast cancer. In recent years, studies have also proposed that there is a complex link among them.^9^^,^^15^

There were several limitations in this study. First, the authors did not further explore the pharmacological and toxicological effects of OXY. Second, the authors speculate that oxycodone promotes the antitumor properties of PTX, but the authors did not intervene with the signalling pathway and autophagy to further explore the specific roles of PI3K/Akt/mTOR and autophagy in the effects of OXY and/or PTX on breast cancer cells. This study investigated the fact that autophagy, EMT and PI3K/Akt/mTOR signalling were participate in SKBR3 cells and regulated by OXY alone or combined with PTX. Meanwhile, the authors believed that OXY can promote the apoptosis of SKBR3 cells and enhance the antitumor effects of PTX *in vitro*.

## Conclusions

The present study indicates that OXY can augment the antitumor effect of PTX on breast cancer *in vitro*. Therefore, PTX in combination with OXY may serve as a potential strategy for the treatment of breast cancer.

## Availability of data and materials

The data that support the findings of this study are available from the corresponding author upon reasonable request.

## Ethical approval

The manuscript conforms to the STROBE Statement. This experiment does not involve humans and animals, and there is no ethical approval.

## Authors’ contributions

Conceptualization: FL; Data curation: FL; Investigation: FL, HY; Methodology: FL, CX; Validation: MM; Funding acquisition: SF; Writing - original draft: FL; Writing - review & editing: SF.

## Funding

The present study was supported by the National Natural Science Foundation of China (grant nos. 81971045) attributed to the Department of Anesthesiology, Women's Hospital of Nanjing Medical University, Nanjing Maternity and Child Health Care Hospital, Nanjing, Jiangsu Province, China.

## Conflicts of interest

The authors declare no conflicts of interest.
